# Shared decision-making and health for First Nations, Métis and Inuit women: a study protocol

**DOI:** 10.1186/1472-6947-12-146

**Published:** 2012-12-18

**Authors:** Janet Jull, Dawn Stacey, Audrey Giles, Yvonne Boyer

**Affiliations:** 1 Institute of Population Health, Faculty of Graduate and Postdoctoral Studies, University of Ottawa, Ottawa, Canada; 2 Ottawa Hospital Research Institute, Clinical Epidemiology Program, School of Nursing, Faculty of Health Sciences, University of Ottawa, Ottawa, Canada; 3 School of Nursing, Faculty of Health Sciences, University of Ottawa, Ottawa, Canada; 4 School of Human Kinetics,Faculty of Health Science, University of Ottawa, Ottawa, Canada; 5 Faculty of Graduate and Postdoctoral Studies, University of Ottawa, Ottawa, Canada; 6 Minwaashin Lodge, The Aboriginal Womens's Support Centre, Ottawa, Canada

**Keywords:** First Nations, Inuit and Métis women, Shared decision-making, Equity, Health equity, Participatory research principles, Cultural adaption

## Abstract

**Background:**

Little is known about shared decision-making (SDM) with Métis, First Nations and Inuit women (“Aboriginal women”). SDM is a collaborative process that engages health care professional(s) and the client in making health decisions and is fundamental for informed consent and patient-centred care. The objective of this study is to explore Aboriginal women’s health and social decision-making needs and to engage Aboriginal women in culturally adapting an SDM approach.

**Methods:**

Using participatory research principles and guided by a postcolonial theoretical lens, the proposed mixed methods research will involve three phases. Phase I is an international systematic review of the effectiveness of interventions for Aboriginal peoples’ health decision-making. Developed following dialogue with key stakeholders, proposed methods are guided by the Cochrane handbook and include a comprehensive search, screening by two independent researchers, and synthesis of findings. Phases II and III will be conducted in collaboration with Minwaashin Lodge and engage an urban Aboriginal community of women in an interpretive descriptive qualitative study. In Phase II, 10 to 13 Aboriginal women will be interviewed to explore their health/social decision-making experiences. The interview guide is based on the Ottawa Decision Support Framework and previous decisional needs assessments, and as appropriate may be adapted to findings from the systematic review. Digitally-recorded interviews will be transcribed verbatim and analyzed inductively to identify participant decision-making approaches and needs when making health/social decisions. In Phase III, there will be cultural adaptation of an SDM facilitation tool, the Ottawa Personal Decision Guide, by two focus groups consisting of five to seven Aboriginal women. The culturally adapted guide will undergo usability testing through individual interviews with five to six women who are about to make a health/social decision. Focus groups and individual interviews will be digitally-recorded, transcribed verbatim, and analyzed inductively to identify the adaptation required and usability of the adapted decision guide.

**Discussion:**

Findings from this research will produce a culturally sensitive intervention to facilitate SDM within a population of urban Aboriginal women, which can subsequently be evaluated to determine impacts on narrowing health/social decision-making inequities.

## Background

Shared decision-making (SDM) is a central feature of patient-centred care [1,2]. SDM approaches have been found to improve clinical decision-making processes and increase health care client satisfaction [3,4]. Client preferences are inadequately addressed in physician – client communication; however, client values and needs for health information can be met through the use of SDM approaches [4].

SDM is enacted through health care delivery approaches and tools, and defined as a process that promotes collaboration between healthcare providers and recipients in decisions affecting health [5,6]. As well as structuring a collaborative, client-centred approach between practitioner and client, SDM promotes the sharing and use of information on the benefits and harms of care options in the form of patient decision aids, which assist the client in making preference sensitive decisions [7]. Patient decision aids complement practitioner counselling; they have been found to increase people’s involvement in making more informed and values-based care decisions [7]. SDM approaches and tools are currently being developed and implemented in international settings and are evolving as standards of care [1,2].

While SDM approaches and tools have been found to be effective in supporting clients to make informed decisions about health, evaluation of these approaches with Aboriginal peoples^a^ has not occurred. In comparison to those living in Canada who are of Euro-Canadian descent, Inuit, Métis, and First Nations peoples experience relatively poor health [8,9], and are recognized as experiencing significant health inequities [10,11]. The health of First Nations, Métis, and Inuit peoples in Canada has been defined as poor, with shorter life expectancies, and higher rates of illness, injury, and suicide when compared to non-Aboriginal Canadians [12,13]. Moreover, Aboriginal women as a population have been identified as being in a far worse predicament. For example, while Inuit, Métis and First Nations populations are very diverse, there are general trends indicating that these groups experience significantly higher rates of chronic disease such as diabetes. Overall, Aboriginal women are more likely than those in the general population [14,15] or Aboriginal men [15], to be diagnosed with diabetes. Further, violence against women is also a significant issue within Canada, and violence against Aboriginal women has been documented as of particular concern [16]. Women in Métis, Inuit, and First Nations communities play important roles as caregivers, community leaders, and keepers of community knowledge; therefore, poor health of Aboriginal women is a significant issue for Aboriginal populations [17]. Aboriginal peoples, and in particular Aboriginal women, must have care that meets their needs – in effect, care that is client-centred.

Meanings of health are unique to individuals and populations, and a consensus on the meaning of the term ‘health’ does not exist [18]; it is therefore important to understand that the perception of decision-making needs for health varies between populations. Decision-making in health settings for Aboriginal peoples requires attention to cultural beliefs not yet evident within Western health and social care frameworks [19]. Little is known regarding the processes or outcomes for Aboriginal women using SDM approaches in care settings or the cultural relevance of SDM approaches or tools such as patient decision aids for Aboriginal populations.

### Objective and research questions

The overarching objective proposed for this study is to explore health and social decision-making needs of Aboriginal women and to engage them in culturally adapting an SDM approach. The study will involve three phases, each focused on one of the following research questions:

1. What are effective interventions to support Aboriginal peoples making health decisions?

2. What are the experiences of Aboriginal women in making health or social decisions?

3. What is the usability of a culturally adapted Ottawa Personal Decision Guide for Aboriginal women?

## Methods

A three phase mixed methods project was designed with the collaboration of Minwaashin Lodge (“the research partner”), located in Ottawa, Canada, and incorporates a postcolonial theoretical framework and principles of participatory research. Minwaashin Lodge is an organization dedicated to providing intervention services and programs to Métis, First Nations, and Inuit women, children and youth who are survivors of family violence and/or the residential school system, including the inter-generational impacts of violence against Aboriginal peoples in Canada.

A postcolonial perspective provides a theoretical lens to show everyday experiences of marginalization of First Nations, Inuit and Métis people that occur in day-to-day relationships and in the systems structuring human relations [20], such as the health care setting [20,21]. Postcolonial theory is particularly relevant for the proposed study, which places Aboriginal peoples as central to a process of change with non-Aboriginal partners [22]. The principal investigator for this study is of Euro-Canadian descent and has collaborated with the research partner using principles of participatory action research to produce this study protocol. The principles of participatory action research (PAR) are emerging in the literature as an appropriate approach for engaging in Aboriginal research partnerships [23], and have been demonstrated as particularly successful in broader, interdisciplinary health promotion initiatives within Aboriginal populations [24], including multilevel interventions [25]. The University of Ottawa Research Ethics Board granted ethics approval for this study in July 2012 (#H05-12-05).

This protocol describes a research study design consisting of three complementary phases. Each phase sequentially informs the next.

### Phase I

Systematic review of Aboriginal peoples and health decision-making.

#### Research Question 1

What are effective interventions to support Aboriginal peoples making health decisions?

#### Background

Most literature exploring concepts relating to SDM, health decisions, and Aboriginal peoples, (internationally referred to as Indigenous peoples^b^), concentrates on describing health decision-making [26-28], health equity issues and the role of Indigenous peoples within cancer care [29-31] or advanced care planning [32-34]. Studies have suggested that patient decision aids can improve decision quality and empower immigrant women to make informed decisions based on personal values [35] or have narrowed the gap between racially distinct groups through engaging clients in a process to make decisions about healthcare services [36]. While interventions that incorporate concepts of SDM have been shown as effective for translating research to inform preference sensitive health decisions [4,7], little is known about interventions to support SDM with Aboriginal peoples.

#### Method/design

A systematic review guided by the Cochrane Handbook [37], and structured to meet the Preferred Reporting Items for Systematic Reviews and Meta-Analyses (PRISMA) [38] criteria was developed. To assist in the development and implementation of the review, a reference group was formed of recognized experts in the area of Aboriginal health issues, information services, decision-making tools and approaches, knowledge translation, systematic review methodology, collaborative research approaches with Aboriginal/Indigenous peoples, quantitative and qualitative methodologies, and library sciences.

Study inclusion/exclusion criteria were framed in a systematic manner, using the elements of a clinical question and include population, intervention, comparator, and outcome (PICO) [39] (Table 1). All studies evaluating interventions for SDM in health decisions with Aboriginal populations will be included: randomized control trials, cross-over, cohort, case controlled studies, and cross-sectional survey.

**Table 1:** Phase I study PICO criteria

*Population*	Defined as Aboriginal and/or Indigenous peoples and as making a health or social decision for themselves and/or a family member. Studies in which Aboriginal and/or Indigenous peoples are included with non-Aboriginal/Indigenous people will be excluded unless the findings for each of the sub-groups are reported separately.
*Intervention*	Any intervention to influence health decision-making.
*Comparator*	Any comparison including usual care.
*Outcomes*	Attributes of the decision [3] and attributes of the decision process [40].
*Publication status*	Published.

### Search strategy

The search strategy protocol was developed collaboratively with an academic reference librarian, after consultation with subject experts on health, decision-making and Aboriginal peoples’. The search will focus on all major databases relevant to the subject matter: MEDLINE, EMBASE, PsychInfo, CINAHL, Proquest Nursing and Allied Health, ERIC, and Cochrane, and will include all years for the database, i.e., from the earliest data sources on each database, e.g., 1947 or earlier, and up to 2012. To supplement the database search, a planned hand search of grey literature for articles on PubMed and checking of reference lists of identified articles for relevant studies will be conducted [41]. As well, a planned hand search of journals found to be associated with the subject of the search will be conducted [37] from one year prior to the review end date.

#### Screening of studies

Following the identification of studies, duplicates will be removed and three levels of screening will be conducted by two independent reviewers, using standardized forms. Level one screening will be a title screen to determine study relevance to the overall objective of the systematic review. All studies identified as “included” and “unsure” will be retained for the level two screening; only studies excluded by both reviewers will be excluded. Level two screening will be a title and abstract screen based on the inclusion/exclusion criteria (PICO). All titles and abstracts identified as “included” or “unsure” by either reviewer will be retained for the level three full text screening; those that are identified by both reviewers as “excluded” will be excluded from further screening. Following level three screening, any disputes between reviewers will be resolved, including those articles identified as “unsure” and resulting in a final group of studies identified by both reviewers as meeting the inclusion criteria. If required, a third person will be available to assist with arbitration. During level two and three screening, rational for exclusion will be documented.

#### Data collection

All included studies will have their data extracted using a standardized form, which will be pilot tested prior to commencing data extraction. During the data extraction process, a second reviewer will verify the accuracy of extracted data. Data will include authors, setting, characteristics of the intervention, study design, characteristics of the participants, and findings relevant to outcomes for this review.

#### Study quality

Two independent reviewers will assess the quality of included studies using the appropriate critical appraisal skills programme (CASP) checklist [42]. If randomized control trials are found, the Cochrane Collaboration Risk of Bias Tool will be used in quality appraisal to examine internal validity [37].

#### Analysis

Characteristics of included studies will be analyzed descriptively. A decision to conduct meta-analysis versus a qualitative synthesis of findings will be determined once data are extracted and the heterogeneity between studies can be assessed. Should it be possible to combine the data between studies to determine an overall statistic on the effectiveness of the intervention on the experimental versus control groups, meta-analysis will be used (i.e., studies demonstrate that similar outcomes were used with similar interventions). A descriptive analysis of data findings will situate study findings within historical and social colonial society [22].

#### Bias

To minimize risk of bias [43] and to ensure that Assessment of Multiple Systematic Reviews (AMSTAR) criteria for methodological quality are met [44], the protocol for the systematic review was established a priori, and plans made for two researchers to screen identified literature, participate in data extraction, and to quality appraise included studies.

### Phase II

Decision-making experiences of Aboriginal women.

#### Research question 2

What are the experiences of Aboriginal women when making health or social decisions?

Specific research questions include: 1) What are the health/social decision-making needs of urban Aboriginal women?; 2) What are the barriers to involving urban Aboriginal women in their health/social decision-making?; 3) What are potential supports to enhance the health/social decision-making experiences of urban Aboriginal women?

#### Methods

An interpretive descriptive qualitative study will be conducted in collaboration with Minwaashin Lodge and with the clients of Minwaashin Lodge (urban dwelling Aboriginal women). As a qualitative approach effective for describing health care events, interpretive description is an inductive analytic approach [45]. For this study the events of interest will be Aboriginal women’s recent experiences in making decisions affecting their health, or the health of someone they care for. The Ottawa Decision Support Framework (ODSF) has informed the study design.

The ODSF is a framework developed to guide people through health and social decisions and involves three key elements: decisional needs, decision support, and decision quality [46]. It is used to describe the hypothesis/assumption that unresolved decisional needs may negatively influence decision quality, and that decision support is aimed at addressing decisional needs. The ODSF can be used to structure the assessment of decisional needs within a range of populations [3] and is suitable for use within this study. The needs assessment interview guide is based on the ODSF and informed by published studies on other populations’ decision-making needs [3,46]. Most importantly, this guide was developed to incorporate information conveyed by members of the research partner, Minwaashin Lodge, who indicated that Aboriginal women prefer opportunities to tell their stories, rather than respond to a highly structured process of questioning.

#### Participants and procedures

Participants will be female clients of Minwaashin Lodge, living in Ottawa and of Inuit, First Nations, or Métis descent, and identified as having recently (i.e., within the past 6 months) been a part of a health/social decision for herself or a family member. They will be asked to participate in an interview conducted in English. Women will be purposefully invited to participate in the study and are anticipated to represent a variety of ages (younger, middle aged, older) and family form (single, with/without children). Recruitment will continue until data saturation plus an additional three participants, to ensure that no new themes are identified. We anticipate reaching saturation with 10 participants, and therefore recruiting a total of 13 women [47].

Individual interviews were selected to support dialogue with the women about their experiences of how decisions affecting health are made with a professional. The women will be invited and informed about joining the study in accordance to Lodge protocol by Minwaashin Lodge representatives and with recruitment posters. After reviewing and signing the consent form with the researcher, participants will be interviewed for 60 to 90 minutes using semi-structured interviews, including a request for non-identifying demographic information. Any questions that the participant does not feel comfortable answering will be omitted. The interviews conducted with the women will be digitally-recorded, transcribed verbatim, and field notes from the researcher will be included as part of the gathered data.

#### Analysis

Demographic data will be entered into Excel database and analyzed descriptively by the researcher. NVivo software [48] will be used during the process to assist with organization of data during analysis. Transcribed interviews will be analyzed using the interpretive descriptive method [45]. The process of qualitative data analysis will use the three step method of data reduction, data display, and drawing conclusions/verification [49]. The data findings, guided by the three research questions, will be organized and evaluated using the postcolonial theoretical lens described by Battiste [22] to contextualize the data, and final findings developed with the Minwaashin Lodge advisory group. This approach to data analysis will support and increase the likelihood that meaningful health decision-making needs, barriers and supports are identified.

Findings from phase II will be used to inform phase III.

### Phase III

Adaptation and usability testing of a culturally relevant patient decision support tool.

#### Research question 3

What is the usability of a culturally adapted Ottawa Personal Decision Guide for Aboriginal women?

#### Design

Cultural adaptation of the Ottawa Personal Decision Guide (OPDG) will be conducted through a collaborative and iterative process between the researcher, Minwaashin Lodge, and Aboriginal women, and then followed by usability testing. The overarching aim of the OPDG adaptation process is to develop a tool defined by women as useful, and to support the fidelity of the culturally adapted OPDG [50].

#### Intervention for adaptation

The OPDG is a tool that has been validated for use in making any health-related or social decisions [51]. It can be used to help people assess their decision making needs, summarize their current knowledge (of options, benefits, and harms), clarify their values associated with option outcomes, plan the next steps, and track their progress in decision making. Although the OPDG has been used in a variety of care contexts and countries outside of Canada (e.g., Japan and U.S.) [52,53] the OPDG has not yet been evaluated for use within Aboriginal populations.

#### Participants – focus groups

Clients of Minwaashin Lodge that live in Ottawa, are of Aboriginal descent, and are able to participate in an English language interview will be purposefully selected by the research partner to participate in focus groups adapting the OPDG. Participants will be women representative of a range of ages and family form and will also be identified as having made a health/social decision in the past six months for themselves or a family member. In studies using focus groups to explore health issues, five to eight participants have been identified as ideal [54,55], and fewer participants identified as more effective for discussion of sensitive subjects [56]. For this reason, five to seven women will be recruited for participation in the OPDG adaptation focus groups.

#### Participants – usability testing

Participants for usability testing will be recruited separately from the focus group cohort, and will be clients of Minwaashin Lodge, living in Ottawa, of Aboriginal descent, and able to participate in an interview in English. The women will participate individually in an iterative process of testing the decision aid adapted by the focus group. As 80% of usability problems have been found within four to five participants [57], five to six participants will be recruited for establishing and refining data during usability testing with decision tools [58].

#### Procedures - focus group

The focus groups will be facilitated by the researcher and a research assistant. They are anticipated to take up to three hours each. Following informed consent by participants, non-identifying demographic information will be collected. Participants will then be guided as a group by the researcher through the use of the OPDG with an example decision. The group will be asked to provide general feedback on the tool (e.g., readability, organization). Then, the tool will be reviewed step by step to discuss specific adaptations to language and/or flow of ideas, and to document a rationale for changes. The focus groups will be digitally recorded and transcribed verbatim, and field notes from the researcher and research assistant will be included as part of the gathered data.

#### Procedure – usability testing

The usability testing will be conducted during a 60 to 90 minute role play in which the OPDG is used to prepare the participant for making a preference sensitive health/social decision. Following informed consent and collection of non-identifying demographic information, the participant will be instructed in the “think-aloud” technique for use during the role play. In the think-aloud technique, participants are instructed to say their thoughts out loud while performing a task, and the interview is digitally-taped then transcribed [59]. The aim of the think aloud technique is to gain an understanding of the women’s views and experiences using the adapted OPDG. During the initial interviews, the think-aloud technique will provide baseline measures for user satisfaction and performance, such as ease of readability and comprehension [58]. Information on the functionality of the design will be conveyed in later interviews [58], and contribute information on the usefulness of the OPDG for assisting women in making health/social decisions.

The women will be asked to bring a decision that needs to be made OR provided with a health/social decision identified by Minwaashin Lodge as commonly experienced by clients. The think-aloud methods will be used while the participant uses the adapted OPDG (concurrent), and immediately after using the adapted OPDG (retrospective) while reflecting on the process of using the OPDG. Previous studies have found that using concurrent and retrospective think-aloud methods produce similar results with different perspectives (e.g., usability issues are more verbalized with retrospective methods and more observed with concurrent methods) [60]. The researcher will use a guide with prompts during usability testing. The role-play interviews will be digitally-recorded, transcribed verbatim, and field notes from the researcher included as part of the gathered data.

#### Analysis

For the focus group analysis, data will be transcribed by the researcher and NVivo software [48] used to assist with organization of data during thematic analysis. Data will be thematically organized to map the adaptation process; additionally, summaries of decisions made about the OPDG adaptation will be developed and analyzed for criteria indicating equivalence between the original and end product [50]. The findings will be reviewed with the Minwaashin Lodge advisory group and any additional information from the review will be incorporated into the adapted OPDG.

For the usability testing analysis, the data from the first two to three women users will iteratively inform further development of the OPDG, meaning that the taped and transcribed data from these initial interviews will be analyzed to provide baseline data and used to reveal major flaws in the OPDG. These findings will be brought back to the Minwaashin Lodge advisory group; based on the outcomes of the findings from the initial interviews any decisions to make changes to the OPDG will be made in collaboration with Minwaashin Lodge. The next two to three users will then use think-aloud methods with the iteratively adapted OPDG. Their sessions will also be taped, transcribed and analyzed to inform further changes to the OPDG. Again, findings will be reviewed by the Minwaashin Lodge advisory group and primary investigator, and, if necessary, changes made to the OPDG.

For focus group adaptations and usability testing, demographic data will be entered into an Excel database and analyzed descriptively. Nvivo software [48] will be used to assist with organization of data during thematic analysis, and data analyzed using the interpretive descriptive method [45]. 'The process of qualitative data analysis will use the three step method of data reduction, data display, and drawing conclusions/verification [49], and contextualized using the postcolonial theoretical lens provided by Battiste [22]. Final findings will be reviewed in collaboration with the Minwaashin Lodge advisory group and/or a town hall meeting, and conclusions developed on the feasibility of Aboriginal women using the OPDG within care settings.

### Strengths and limitations

Common limitations in the use of interpretive descriptive methods include: the belief that it is possible to fully comprehend the experience of research participants; seeking to confirm conclusions too early in the analytic process; failing to move beyond merely describing phenomenon within the study; and the researcher attributing significance to something that is not significant to the participants [45]. Despite these potential limitations, during the phase II study, the interpretive descriptive approach creates an opportunity to generate links within the data about women’s experiences, and thereby facilitate new understandings of urban Aboriginal women’s experiences with health decision-making. The phase III study will lead to understandings about how the OPDG may be used by urban Aboriginal women in making decisions that affect health. Additional strengths of the phase III study include maintaining the principles underlying the OPDG and therefore the fidelity of the culturally adapted tool [50]. As well, the study design incorporates the socio-cultural context of the potential OPDG users into the process of adaptation and usability testing, an important feature identified in cross-cultural questionnaires [61].

For both the phase II and III studies, the primary investigator will use strategies to facilitate the evaluation of data synthesis and credibility processes and demonstrating an awareness of how the study findings may potentially be transferred, including: rich, thick description, journal keeping, member checking, an audit trail, identification of clear outcomes, contextualization of study findings.

### Ethics

This research protocol has been designed to support a research agenda respectful of the diverse needs of a population of urban Inuit, First Nations and Métis women. Within Aboriginal contexts, research ethics reflect views on ethical conduct as an integral part of a life which is interdependent with all living and material things. The Tri-Council Policy Statement (TCPS)[62] and Ownership, Control, Access and Possession (OCAP)[63] strive to reflect the unique ethical issues involving research with Aboriginal peoples, and were used in the development of this protocol.

OCAP principles identify and implement the inherent right of self-determination by Aboriginal communities within research studies, and are applicable to all stages of the research process. The principles of OCAP have been acknowledged and supported throughout the development of this protocol (Table 2). The research process has been built upon meaningful engagement and reciprocity between the principal investigator and her thesis committee, and Minwaashin Lodge, and is demonstrated in a letter of support from Minwaashin Lodge for the research process. Participatory research principals and postcolonial theory also contribute to support the principles expressed in OCAP.

**Table 2:** Principles of OCAP [64] and study initiatives

**OCAP principles**	**Study initiatives**
Ownership: An Aboriginal community or group owns information collectively in the same way that an individual owns their personal information.	Minwaashin Lodge is recognised as a full research partner by the University of Ottawa Research Ethics Board.
Control: Aboriginal communities/representative bodies are within their rights in seeking to control all aspects of research and information management processes that impact them.	Key stakeholders and Minwaashin Lodge have been included during development of the three-stage study protocol, and will be co-producers of knowledge during data collection, interpretation, and dissemination.
Access: The right of Aboriginal Peoples to information and data about themselves and their communities, as well as a right to manage and make decisions regarding access to their collective information.	Collected data is to be stored in a mutually agreed upon way to ensure the privacy and confidentiality of participants; the data sets will be accessible by representatives of the Minwaashin Lodge.
Possession: Possession, or stewardship, of data is a mechanism by which ownership can be asserted and protected.	The primary investigator and Minwaashin Lodge act in a collaborative manner e.g. creating opportunities for: meetings, informed questions about the study procedures, on-going email and in-person contact for dialogue. Data will be disseminated in collaboration with Minwaashin Lodge and to stakeholders identified and/or approved by Minwaashin Lodge.

Additionally, the primary researcher was invited by Minwaashin Lodge to participate in Minwaashin’s Culture program and oriented to the Oshki Kizis shelter for women and children to gain a deeper understanding and knowledge of the people providing and seeking services at Minwaashin Lodge. Prior to initiating the research study, the primary researcher attended the Annual General Meetings and weekly culture events, as well as special educational initiatives, and including meals at Minwaashin Lodge. These opportunities allowed the primary researcher to meet with clients, Board members, program leaders, and other volunteers, and to become acquainted with the research partner. An on-going process of relationship building between the primary investigator and Minwaashin Lodge has resulted in the development of a protocol designed to be inclusive, meaningful, and respectful and in compliance with OCAP principles.

## Discussion

There are few studies of interventions influencing the health status of Aboriginal populations [65]. This protocol outlines a strategy for urban Aboriginal women to develop a resource that can influence their participation within health and social systems. The study will make four significant contributions: 1) it will synthesize studies describing effective interventions to support Aboriginal people making health decisions; 2) explore and describe the health/social decision-making needs of a group of urban Aboriginal women; 3) create a culturally relevant tool to facilitate SDM for urban Aboriginal women in health/social care settings and; 4) evaluate the feasibility of an intervention (the OPDG) designed to narrow the health equity gap experienced by Aboriginal women. Moreover, the study has been designed with a focus on an integrated knowledge translation and dissemination strategy.

This protocol is the result of research partner and researcher collaboration. It was developed to meet the needs of the knowledge users to and engage the research partner and other key stakeholders throughout the research process. It can therefore be considered an integrated knowledge translation (KT) project [66]. Integrated KT describes an iterative cycle of knowledge acquisition and implementation; the results of this study will inform further development of decision tools and approaches to facilitate Aboriginal women in identifying and achieving goals they identify for SDM within health and health related settings. In conclusion, this protocol describes a study supporting development of community-research partnerships, and development of tools with the potential for promoting equity for Métis, First Nations and Inuit women in Canadian care settings.

## Endnotes

^a^ “Aboriginal Peoples” is a collective name for all of the original peoples of Canada and their descendants. Section 35 of the *Constitution Act, 1982* specifies that the Aboriginal Peoples in Canada consist of three groups – First Nations, Inuit and Métis. It is not used to describe only one or two of the groups (65).

^b^ “Indigenous peoples” is an international term and refers to people who:

Identify themselves and are recognized and accepted by their community as Indigenous.

Demonstrate historical continuity with pre-colonial and/or pre-settler societies.

Have strong links to territories and surrounding natural resources.

Have distinct social, economic or political systems.

Maintain distinct languages, cultures and beliefs.

Form non-dominant groups of society.

Resolve to maintain and reproduce their ancestral environments and systems as distinctive peoples and communities.

(United Nations Permanent Forum on Indigenous Issues, 2006) (66).

## Competing interests

The authors declare that they have no competing interests.

## Authors’ contributions

JJ is the primary investigator, conceived, led and co-ordinated the development and writing of the manuscript; DS, AG, YB and ML participated throughout the development and writing of the manuscript by contributing intellectual content and feedback on drafts of the manuscript. All authors read and approved the final manuscript.

## Funding

Partially funded by KT Canada.

## Pre-publication history

The pre-publication history for this paper can be accessed here:

http://www.biomedcentral.com/1472-6947/12/146/prepub
